# “Sacred ground for kids”: Institutional perspectives on rural school‐based health centers as patient‐centered medical homes

**DOI:** 10.1111/jrh.70098

**Published:** 2025-12-20

**Authors:** Xue Zhang, Mildred E. Warner

**Affiliations:** ^1^ Department of Biobehavioral Health The Pennsylvania State University University Park Pennsylvania USA; ^2^ Department of City and Regional Planning Cornell University Ithaca New York USA; ^3^ Department of Global Development Cornell University Ithaca New York USA

**Keywords:** child empowerment, confidentiality, patient‐centered medical home, rural health, school‐based health center

## Abstract

**Background:**

School‐based health centers (SBHCs) can function as patient‐centered medical homes (PCMHs), but few studies examine how SBHCs fit the PCMH definition and address the challenges of rural health disparities among children and adolescents.

**Methods:**

Note that 12 semi‐structured interviews were conducted in four rural counties in New York State with school superintendents, SBHCs medical providers, and health care network administrators and between January 2024 and April 2024. Participants were identified using snowball sampling. Interviews were transcribed. Framework analysis was applied with thematic coding based on the PCMH framework. NVivo 14 was used to generate the final set of themes.

**Findings:**

Our analysis confirms SBHCs fit the PCMH model—accessibility, comprehensive, family‐centered, coordinated, continuous, and compassionate care—to improve health care access for rural children and empower children to advocate for their own health. We identify privacy and confidentiality as additional important elements in the PCMH model, which ensure children's empowerment. However, they present special challenges for rural SBHCs. Addressing these challenges requires attention to information sharing between SBHCs and schools and the need for trust and communication to empower children, while not alienating school partners and parents. This may explain why so few rural SBHCs are PCMH.

**Conclusions:**

This qualitative thematic analysis shows SBHCs can serve as PCMHs in rural communities. It also highlights the importance of privacy, confidentiality, trust, and communication between SBHCs, schools, parents, and children.

## INTRODUCTION

Children living in rural communities face greater challenges in accessing health care services and have worse health care outcomes.^1^, ^2^, ^3^, ^4^ School‐based health centers (SBHCs) present a practical solution for enhancing health care access among underserved children and youth.^3^, ^5^ SBHC is defined as a clinic located on or near schools that provides at least primary care to children during school hours.^6^ The most common SBHC delivery model is the traditional school‐based approach, where students access care on campus.^7^ Based on the most recent 2022 national SBHC census, 92% of SBHCs use this approach, 4% use a school‐linked model that partners with nearby health care facilities, and 3% use mobile units parked on or near school grounds.^7^ Telehealth is widely used, with 90% of surveyed SBHCs providing some services through telehealth.^7^

Through joint efforts between schools and qualified health care providers, SBHCs typically offer preventive care, behavioral health support, acute care, and chronic condition management, thereby promote children's and adolescents’ health care access, educational outcomes, and individual and community well‐being.^5^, ^8^, ^9^, ^10^, ^11^ As of 2022, there are approximately 3900 SBHCs in the United States.^7^ While historically concentrated in urban centers, the number of SBHCs is increasing in rural communities, with 36% of SBHCs located in rural schools.^5^ However, most research on SBHCs remains urban‐focused, leaving a research gap regarding the role of SBHCs in rural communities.^9^, ^12^, ^13^

SBHCs have the potential to operate as patient‐centered medical homes (PCMHs), aligning with the core principles of accessibility, continuity, comprehensiveness, family‐centeredness, coordination, and compassion.^14^, ^15^, ^16^, ^17^, ^18^ However, SBHCs encounter challenges in fully functioning as PCMHs due to operating constraints tied to the school year, limited resources attributed to their scale, and financial sustainability issues.^17^ Additional challenges come from the SBHC model itself, which is built on the school‐health care–patient partnership rather than the traditional health care–patient PCMH model. Differences in organizational regulations and priorities between schools and health care systems can create challenges related to confidentiality and privacy.^15^, ^19^, ^20^ Moreover, patients may encounter access barriers, as in many states, only enrolled students are eligible to use SBHC services, excluding preschool‐aged children and other family members from care.^7^ This limits opportunities for family‐centered care. The difficulties that SBHCs face in embodying the PCMH model were demonstrated in a study examining the 2013–2014 National Census of School‐Based Health Centers, which found that only 29% of SBHCs had any type of recognition as a PCMH and only 17% received national‐level recognition.^21^ Previous studies evaluating SBHCs as PCMHs are based on surveys at the national level,^21^, ^22^ and surveys focused on parents’ and adolescents’ perspectives in an urban setting.^14^ This study is the first to explore institutional perspectives from school superintendents, SBHC medical providers, and health care network administrators on how SBHCs function as PCMHs in rural communities. We examine challenges in SBHC enrollment, especially from limited anonymity in rural settings.

## METHODS

### Study area

We used exploratory methods to examine institutional perspectives on SBHCs across four rural counties in New York State: Delaware, Schoharie, Chenango, and Otsego. Chenango, Otego, and Delaware counties are categorized as nonmetropolitan counties with urban population of 20,000 and fewer.^23^ Although Schoharie county is in the capitol region metro area, the county only has 29,970 population of which only 5228 are urban, based on the American Community Survey 2018–2022,^24^ and the school district with the SBHC does not have an urban area.^25^ Schoharie county is historically viewed as a rural county in New York State.^26^ All four counties have a shortage of primary care physicians.^27^

The Bassett Healthcare Network serves as the main health care provider in the region. As of 2023, Bassett operated 21 SBHCs across 17 of 38 school districts in these counties, with the first SBHC established in 1992. Bassett SBHCs use the traditional school‐based approach, and provide comprehensive health care, preventive dental services, and mental health services at school, as well as telehealth for some specialty services.^28^ In 2023, 81% of students in these 17 school districts were enrolled in the SBHC. Bassett's SBHCs have received recognition from the National Center for Quality Assurance as PCMHs.^29^

### Instrumentation and procedure

This study explores how SBHCs function as PCMHs and the challenges and opportunities they encounter in rural communities. Data were collected from 12 semi‐structured interviewers, involving four school superintendents, five SBHCs medical providers, and three administrative employees from the Bassett Healthcare Network. Participants were identified using snowball sampling, starting with a pilot interview with the Bassett SBHC manager, who then recommended additional interviewees. In each county we interviewed at least one school superintendent and at least one SBHC medical provider.

This study was exempted by the Cornell Institutional Review Board. Consent forms were sent to interviewees before the interviews, and oral consent was obtained before recording. The interviews were conducted between January 2024 and April 2024, each lasting 60 min.

To evaluate the role of SBHCs in promoting children's health care utilization in rural communities, we asked interviewees the following:

1. Why does your school/program support the SBHC?
2. What do you see as the most important elements/challenges in school‐SBHC collaboration?
3. What do you think is the impact of the SBHC on children's health?
4. What barriers to access does the SBHC help address?

Then, we explored the challenges and opportunities faced by SBHCs in rural communities by asking:

5. What are some barriers that cause some parents/students not to use the SBHC?
6. What is your vision for the SBHC?

### Data analysis

Each interview was recorded via Zoom, with notes taken and discussed immediately afterward. During team discussions, we noticed that all three Bassett administrative employees emphasized “patient‐centered medical home (PCHM)” as the core of the organization. After reviewing interviews with school superintendents and SBHC providers, we concluded that the PCMH framework effectively captured the role of SBHCs in rural communities. Then, transcripts were cleaned, and we applied PCMH framework analysis primarily to interview Questions 1–4. We used deductive thematic coding based on seven predefined PCMH categories from the American Academy of Pediatrics (accessible, comprehensive, coordinated, family‐centered, continuous, compassionate, and culturally effective).^30^ We also conducted inductive thematic analysis to examine the challenges and opportunities faced by SBHCs in rural settings (interview Questions 5 and 6). This analysis allows us to identify elements that may be underrepresented in the PCMH model. The list of codes and themes was then discussed and disagreements resolved within the research group. For example, we initially applied all seven predefined PCMH categories but found that interview content related to family‐centered care (e.g., “working with families every step of treatment”) overlapped with coordination with parents and family, while culturally effective (e.g., “SBHC is sacred ground for kids”) aligned with compassionate care. To maintain the focus, we incorporated family‐centered and culturally effective elements into the coordination and compassionate care categories, respectively. Finally, a lead researcher employed qualitative software (NVivo 14) to deductively code the interviews, resulting in a final set of themes.

## RESULTS

Qualitative analysis of the 12 interviews from school superintendents, SBHC medical providers, and health care network (Bassett) administrators identified three major themes. Detailed illustrative quotes from the three stakeholder groups are shown in the Appendix. We selected one quote from each type of stakeholder across all themes and most subthemes. Appendix illustrates that thematic saturation was reached. Each theme includes responses from all three types of stakeholders, but with some different emphases on subthemes. School superintendents and SBHC medical providers most frequently discussed how SBHCs function as PCMHs (Theme 1), with schools emphasizing accessibility, SBHC providers focusing on care delivery and coordination, and Bassett highlighting the overarching mission of SBHCs. Privacy and confidentiality (Theme 2) were discussed primarily by school superintendents and Bassett administrators, with schools providing more perspectives from parents and families. Theme 3 on challenges of SBHCs serving as PCMHs includes substantial input from SBHC medical providers. A detailed breakdown of each theme is shown below.

### Theme 1: SBHCs fit the PCMH model

#### Accessibility and comprehensive care are key features of PCMHs in rural SBHCs

Accessibility and comprehensive care, as the primary indicators of a PCMH, are important features of rural SBHCs according to interview findings. All four school superintendents mentioned that SBHCs provide reliable health care that students typically would not access otherwise. We found that SBHCs play an important role in administering immunizations, including Human Papillomavirus (HPV) vaccines and flu shots. One SBHC medical provider noted “the HPV vaccination rate has more than doubled year over year, and 20% of students got flu shots this year.” SBHCs also enhance children's access to dental health and mental health, addressing health disparities in rural communities that are often exacerbated by financial constraints and physician shortages. Some SBHCs have implemented telehealth to expand health care accessibility.

Beyond facilitating access, SBHCs improve health care quality. Interviewees mentioned that SBHCs are the most reliable and consistent form of health care for rural children. One SBHC medical provider noted “I feel like we have a little more flexibility here at school‐based health and are able to get kids in the same day or next day at the latest for any acute illness or injury.” These characteristics highlight the advantages of SBHCs in health care access when compared to other pediatric clinics in rural communities.

Rural regions often grapple with limited health care. Bassett SBHCs have removed the access barriers due to geographical isolation and health care cost by providing comprehensive health care within school at no cost. School superintendents noted “In a rural community, access to health care, including mental health, dental health, physical health, is very challenging. Just because our geographic location presents access barriers. Ease of access [provided by SBHCs] is the biggest thing and no cost [to parents] is huge.” SBHCs remove the treatment barriers, and underserved rural children and adolescents receive health care that would otherwise not be accessible to them. One SBHC provider stated, “We hired an administrative assistant who came from the local pediatrics office… About a month into her tenure…she said ‘I thought we saw everybody. But there's another level of kids that we were not even serving, that we never see—that you're getting here at school‐based health’.”

SBHCs mitigate transportation barriers for families in rural communities. By situating health centers in schools, children can access health care without significantly disrupting their instruction time. Additionally, children have the autonomy to visit the health center independently. This eliminates the need for parental accompaniment or additional transportation arrangements, allowing parents to remain at work.

#### Rural SBHCs emphasize coordinated care

Coordinated care is a cornerstone of the PCMH model and is also an essential component in Bassett's SBHCs. The Bassett PCMH model is built on coordination among SBHC medical providers, school nurses, teachers, school social workers, school administrators, and parents. School superintendents mentioned the close relationship between SBHC practitioners and the school nurse, and their ability to collaborate on immunization and all the aspects of preventive care. Interviewees also provided examples of SBHCs engaging in coordinated efforts with teachers regarding children's needs, with school administration to disseminate vaccine information, and partnering with social workers and parents to address children's broader needs. This integrated approach enables SBHCs to deliver family‐centered care throughout the entire health care process. One school superintendent noted that SBHCs help keep parents at work, schedule appointments, and enroll in Medicaid if needed. A Bassett administrative employee gave an example of the collaboration between Bassett, SBHC medical providers, social workers, and parents for a child who needed a kidney transplant. The SBHC and social workers worked together to make sure the child got to appointments, educated parents on the child's special diet, closely communicated with the parents, and the child's health care cost was covered by the SBHC.

#### Continuous and compassionate care empowers underserved rural children

Continuous and compassionate care is central to empowering children within the Bassett PCMH model. SBHCs in New York State exclusively serve students enrolled in their respective schools. Interviewees emphasized how SBHCs empowered children to advocate for their own health care needs and seek out services independently. One school superintendent stated “We'll get high school students who can advocate for themselves… they can actually go to the clinic and say, ‘Can I see someone? I'm not feeling well’ versus having to utilize a parent.” SBHCs foster a culture of health by helping students develop a health pattern for preventive care. Interviewees indicated that children who utilize SBHCs are more likely to use more health care services and maintain healthy habits into adulthood, potentially averting chronic diseases like obesity and diabetes. Ultimately, the health patterns established through SBHCs can contribute to the development of healthier adults.

SBHCs are viewed as trusted providers. Compassion from Bassett employees and medical providers builds trust, emotional resilience, and self‐esteem among students. Described by a Bassett employee as a “true PCMH model,” Bassett's SBHCs prioritize improving health care metrics and quality in the community. SBHCs provide a safe space for students to express their emotions, enabling them to address health challenges and develop emotional resilience. Bassett employees affirmed that SBHCs are “sacred ground for kids,” where they “tell us everything, I mean, much more than their parents want to tell us sometimes.” Through providing compassionate care, SBHCs build a supportive environment for all students, especially those lacking caring adults in their lives, thus empowering self‐worth and confidence. One medical provider emphasized the SBHC role in striving to provide a consistent, caring, adult figure that helps children navigate toward a better adulthood. “I asked every staff who interacts with kids, to take on 2–3 kids who need extra attention and make them your special kids…Many of our kids do not have caring adults in their households. I called it ‘the power of one safe caring adult’.”

### Theme 2: Privacy and confidentiality are additional key PCMH features to ensure child empowerment

We found that confidentiality and privacy are necessary for SBHCs to function as PCMHs to empower students to advocate their own health care needs. Confidentiality creates a clear boundary between the school and SBHCs on information sharing about students. Although the confidentiality provided by SBHCs could sometimes conflict with school officials’ desire to understand and coordinate care and education services, the school and the SBHC must build mutual understanding regarding information sharing. Clear lines are needed to ensure student privacy. One school superintendent noted “So there are laws where, medically, they [SBHCs] can't share things about patients, and there are laws for school districts that we can't overshare. So the only time that there would be sharing, I think [is] when the health and safety of a student is at risk and we're needing to support each other with the information. So yes, I would say, there are natural built‐in privacy laws for both sides of us, and I don't think that that barrier gets crossed.” A Bassett administrative employee also noted “They [Schools] are giving us information and we'll just say, we'll look into it…they [schools] don't have access to our records by any means… So, we are tight on confidentiality… more information is coming to us than leaving us.”

Confidentiality can ease parents’ worry on potential stigma that could lead to judgment and differential treatment for students and parents alike. One school superintendent noted that “… for parents, maybe, who are not running their household in the way that they should, there's concern that schools are going to find out information through the clinic about them, and make judgments or treat them or their children differently.” Confidentiality in the SBHC can result in higher SBHC enrollment.

Privacy can empower children to take health care actions, especially on vaccine and contraceptive care. New York State law allows children over age 12 to consent to reproductive health care, certain mental health services, alcohol and drug services, and sexual assault treatments.^31^ This gives students the opportunity to advocate for their own health care needs independently without utilizing a parent. However, our interviewees report that privacy issues deter some parents from enrolling their children in SBHCs. Interviewees highlighted that some parents request full disclosure of their children's medical care and object to contraceptive services offered by SBHCs. A SBHC provider stated “They [parents] don't want them [their kids] to receive those services necessarily without them [parents] knowing.” A school superintendent also noted “They [SBHCs] supply contraceptives. That is a piece that's part of comprehensive health for teens and not all of our families agree with that. So I know that some families have not joined purely for that reason.” To address this challenge, schools assist with communication between parents and SBHCs to improve health care literacy. For example, a Bassett administrative employee noted how the schools help SBHCs reach parents and receive consent. “We [SBHCS] are always looking from the schools for kind of outward endorsements of the SBHC services, right? So just that they [schools] are encouraging parents, in many cases, they [parents] know the school better than they know us if we're relatively new in the community. So we're looking for them [schools] to do that.”

### Theme 3: Challenges in SBHCs serving as PCMHs

#### SBHCs restricted from serving families

The vision for SBHCs emphasizes continuity, comprehensiveness, and family‐centeredness, echoing key PCMH attributes. However, under New York State Law, only enrolled students have access to SBHCs. This creates challenges for continuity of care and family‐centered medical services. One school superintendent highlighted the difficulty of accessing health care after students graduate. Numerous interviewees mentioned their desire for expanded health care services and providers, particularly in mental health and specialist care. They envisioned an SBHC which could provide long‐term care from birth to adulthood. One school superintendent mentioned “for me it'd be awesome if Bassett could see birth through adult… and maybe adults for very basic things.” Interviewees also envisioned SBHCs expanding their services to treat family members and provide more family centered care. SBHC medical providers mentioned “More family‐centered care… I would love to see evening hours for family therapy… that's what would serve families best. There's a lot of things that can't be solved by just talking with the child… The medical home model is family centered as well. So why not integrate the family into the care plan in general?”

#### Loyalty to existing pediatricians is a primary barrier to SBHC enrollment

Enrolling in SBHCs presents several challenges, with loyalty to existing practitioners being a primary barrier, as some parents, especially in small rural communities, are reluctant to have their child(ren) leave the local practitioner. One SBHC medical provider noted “I think people love their pediatrician…even though they never see him. Because the idea of doing that [switching to the SBHC] is sort of a betrayal.” However, there is no conflict between seeing local pediatricians and SBHC medical providers. Bassett SBHCs help address this challenge by letting parents know they can use both the SBHC and their local pediatrician.

#### Lack of funding and resources

Many interviewees mentioned SBHCs need more funding and resources, including more social workers, health care workers, physical space, and continuous education for physicians. More mental health services was the most common demand mentioned by interviewees. One school superintendent mentioned, “We need more help for our young people, especially coming out of COVID‐19, so ideally a psychologist.” Interviewees proposed innovative strategies including implementing electronic consent forms, and integrating more mental health and community services into the clinic. One SBHC medical provider noted “I would like to try and integrate more community services inside of our clinic. Bring the care to the patient.”

## DISCUSSION

**FIGURE 1:**
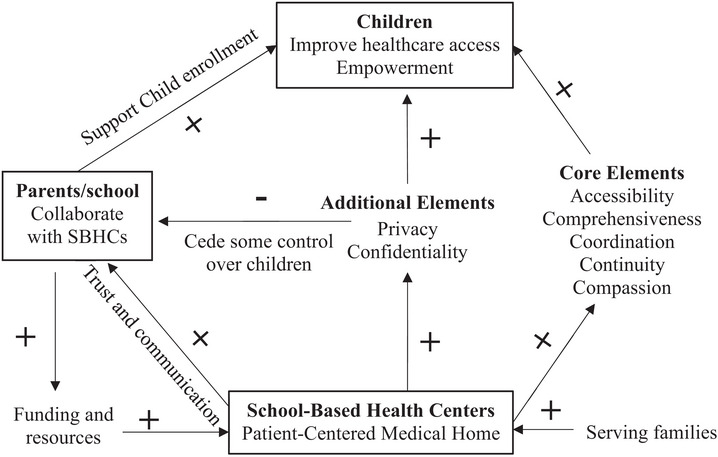
Key elements in school‐based health centers as patient‐centered medical homes.

SBHCs serve as vital health safety nets in rural communities. Among all the elements of PCMH, accessibility is the most commonly achieved, as SBHCs directly address cost and transportation barriers.^9^, ^32^, ^33^, ^34^ Our study shows the Bassett SBHCs include core elements of a successful PCMH model, providing accessible, comprehensive, coordinated, continuous, and compassionate care to improve children's health care access (Theme 1, Figure 1). We also find that confidentiality and privacy are additional key features needed for PMCHs to empower children as advocates for their own care (Theme 2, Figure 1). These are critical, as one medical provider stated, “confidentiality is key.” However, privacy and confidentiality may cause schools and parents to be hesitant about collaborating with SBHCs, as child empowerment may undermine their control (Theme 2, Figure 1). Another challenge to SBHC enrollment is parents’ loyalty to their existing pediatricians (Theme 3). Building trust and communication with parents, and coordinating with schools is essential to mitigate parent concerns (Figure 1). Support from schools and parents can help increase funding and resources for SBHCs. Improving funding and resources and expanding services to family members are important for strengthening SBHCs’ role as PCMHs (Theme 3, Figure 1).

While empowerment is not an explicit part of the PCMH, it is a critical role played by SBHCs.^35^ Lack of confidentiality can limit health care access,^36^ especially for rural children around sexual health.^37^ Our analysis shows this empowerment encourages students to assert independent control over their own health care needs, but it may sometimes conflict with parental authority over children's health, particularly regarding vaccines and contraceptive care. The confidentiality provided by SBHCs also can sometimes conflict with school officials’ desire to understand and coordinate care and education services. However, privacy requires there be clear communication boundaries between schools and SBHCs.

Previous research indicates that concerns about confidentiality and privacy are barriers to SBHCs,^15^ and to rural children's access to care.^36^, ^37^ This may explain why so few SBHCs are PCMH in rural communities. Lack of anonymity is a common characteristic of rural communities that rural health care providers must navigate. It can lead to stigma and reluctance to access care, by adults and children alike.^37^, ^38^, ^39^ Ensuring confidentiality for SBHCs and privacy for children is fundamental, but SBHCs must work to ease the concerns of parents and school officials. For this, communication and trust building are key.

Communication helps parents understand what role SBHCs can play, and schools can be a mediator to support communication between parents and SBHCs. Promotional strategies, such as showcasing SBHCs during school events or parent–teacher meetings, can effectively raise awareness about the benefits and availability of SBHC services and schools can assist with the consent forms, as they regularly communicate with parents. Schools also can help resolve conflicts. One school superintendent described solving a disagreement with parents who deemed a recommended treatment unnecessary and complained to the school. The school helped diffuse the situation by staying in communication and actively reengaging the family. The school superintendent noted we “partner with the family in a problem‐solving solution focused approach. … we [school and SBHC] could have easily retreated back into our own silos and become defensive and exchange blame. But that doesn't get anybody anywhere. And ultimately it was resolved, and it changed some practices on both sides [school and SBHCs].” Communication also helps school officials understand the limits to information sharing and the need to balance the school's desire to support children's health and well‐being with SBHC confidentiality requirements.

SBHCs prioritize case management, but the ability of SBHCs to provide truly family centered care is limited, because in states like New York, SBHCs are not allowed to serve parents or siblings. Nationally, approximately a third of SBHCs serve other community members, 47% serve students’ family members, and 59% serve school staff.^7^ In our case study, we find SBHCs’ role as primary health care providers and “sacred ground for kids” may, ironically, limit their potential as a broader community resource, as this isolates their focus to the children. By contrast, schools have a broader community reach than the SBHCs. Schools often serve as anchor institutions in rural communities, linking social, physical, financial, and institutional aspects of community development.^40^, ^41^ This enables collaboration with other community agencies,^42^, ^43^ and the potential to address broader social determinants of health.

This qualitative study shows the important role of SBHCs in rural communities providing health care for underserved children and adolescents. The perspectives from school superintendents, SBHCs medical providers, and health care network administrators point to several considerations for the practice of SBHCs as PCMH:

1. Confidentiality and privacy are key. SBHCs have a special role to play with children and adolescents—to empower them to take control over their own health care decisions. Additional critical elements of the PCMH model for SBHCs are the privacy and confidentiality they offer children.
2. Communication with parents and schools can build trust. Miscommunication and misinformation are the main barriers to SBHC enrollment. Schools and SBHCs can host information sessions during school events to raise awareness and encourage SBHC enrollment. They must build trust, so that parents and school officials alike understand the need to respect the confidentiality and privacy requirements SBHCs face as health care providers.
3. Expand access to families. Rural communities are often “healthcare desserts.” If SBHCs could extend their services to family members, school staff, and other community members, then SBHCs could potentially function as community health hubs. Achieving this requires policy change at the state level, and recognition of the conflict between SBHCs as “sacred places for kids,” and the broader reach of other PCMH that serve the full range of community members.

### Limitations

This study has several limitations. First, it is a case study focused on four rural counties, which may limit the generalizability of the results to other settings. Second, all the SBHCs included in the study are designated as PCMH, which offers insights into the success of SBHCs within this model, but may not be applicable to all SBHCs. Third, the study examines the institutional perspective on SBHCs, without including opinions from children, families, and other community organizations. Fourth, the study exclusively focuses on rural New York State. Our findings suggest that expanding SBHC services beyond enrolled students to include families and other residents could improve community health in our study site. This expansion would require policy changes at the state level, specifically from New York State Department of Health. However, this state‐specific focus is also a limitation. SBHC policies differ across states, which may affect the generalizability of our findings. Future research could address these limitations by conducting comparative studies with a broader range of SBHCs and gather perspectives from diverse stakeholders to provide a more comprehensive understanding of the benefits and challenges of SBHCs in rural settings.

## CONCLUSIONS

From the perspectives of school superintendents and SBHC health care providers, SBHCs in rural communities play a crucial role in addressing rural health disparities through the PCMH model. By removing barriers to health care access, SBHCs improve access and quality of health care for children in rural areas. In addition to offering comprehensive health care, SBHCs empower children to engage in health care services by providing accessible, comprehensive, compassionate, coordinated, and continuous care. Concerns about privacy and confidentiality may create barriers for schools and parents, but they are critical for children's empowerment. Addressing these challenges requires communication and trust building with schools and parents so they recognize the limits to information sharing with medical providers. This is tricky terrain, but it can be negotiated. For more SBHCs in rural communities to become PCMHs requires special attention to issues of trust, confidentiality, and privacy, and how this affects the relationship between SBHCs, their school hosts and parent partners, and most importantly, the children.

## CONFLICT OF INTEREST STATEMENT

The authors declare no conflicts of interest.

## ETHICS STATEMENT

This study was approved and declared exempted by the Cornell Institution Review Board IRB0148254.

## Supporting information



Supporting information

## Data Availability

Research data are not shared.
