# Supplementary material for: “Sacred ground for kids”: Institutional perspectives on rural school‐based health centers as patient‐centered medical homes
Source: J Rural Health. 2025 Dec 20;41(4):e70098. doi: 10.1111/jrh.70098 (PMC12717837; doi:10.1111/jrh.70098)
Supplement: Supplementary file 1 — Supporting information [file JRH-41-0-s001.docx]

**Appendix 1 Rural SBHC as PCMH: Subthemes and illustrative quotes**

| **Theme 1a: SBHCs Fit the PCMH Model – Accessibility and Comprehensive Care** | Source |
| --- | --- |
| **Comprehensive care:** |  |
| *Preventive care:* In terms of benefits, I think [it is] having reliable access to preventative health care that they typically wouldn't access at all. | School |
| *Vaccinations:* HPV vaccination rate has more than doubled year over year, and 20% of students got flu shots this year. | Bassett |
| *Dental health:* Probably a lot of families don't even have dental coverage and so they've been able to get those cleanings through the district as well as through the school-based health plan. | School |
| *Mental Health*: [We are] able to provide regular counseling service to a pretty large group of kids that really helps families. | SBHC |
| *Telehealth:* They also employ telehealth services where that is necessary. | School |
| *Urgent care:* There’s really not much urgent care outside of the school system. So, it [SBHC] provides opportunities [convenient care]. | School |
| **Improve healthcare quality:** |  |
| I think for most, for many of our students, this [SBHC] is the most reliable and consistent form of health care available to them. | School |
| I feel like we have a little more flexibility here at school-based health and are able to get kids in same day, or next day at the latest, for any acute illness or injury. | SBHC |
| **Remove access barriers due to geographic isolation:** |  |
| In a rural community, access to health care, including mental health, dental health, physical health, is very challenging. Just because our geographic location presents access barriers. Ease of access [provided by SBHCs] is the biggest thing and no cost [to parents] is huge. | School |
| School based health care is essential to help provide needs in some areas that don't have a doctor's office. | Bassett |
| All of the services that we provide here at the school-based center so readily [available] based on the geographic location. | SBHC |
| **Remove access barriers - no cost to families:** |  |
| So I think just that ease of access is the biggest thing and no cost is huge. | School |
| Obviously, the fact that they [children and family] don't have to pay any extra for the services…just to have that care that they don't have to pay out of pocket is amazing. | Bassett |
| There’s no out-of-pocket costs to the kids. We do take insurance, but then co-pays, deductibles, none of that gets billed to the families. | SBHC |
| **Remove treatment barriers:** |  |
| We hired an administrative assistant who came from the local pediatrics office... About a month into her tenure…she said ‘I thought we saw everybody. But there’s another level of kids that we were not even serving, that we never see– that you’re getting here at school-based health. | SBHC |
| **Address transportation barriers:** |  |
| We do have some families in our district that don't have transportation. When a parent can't get them to a facility, it's [SBHC] a great way for their child to get health care. | School |
| People have a hard time even just with putting gas in their car to drive 20 minutes to go to a primary care. I think that we are providing this service to kids that otherwise wouldn't have the means to get to it. | SBHC |
| **Theme 1b: SBHCs Fit the PCMH Model: Coordinated Care** | Source |
| **Collaborative culture:** |  |
| In a place [SBHC] where that psychologist can collaborate with our school counselors, our school psychologists and our school social worker - It mitigates the time. | School |
| We're constantly collaborating with their counseling department. We collaborate with the school nurses and their teachers. And sometimes, it's all kinds of different combinations. | Bassett |
| **Coordinate with school nurses:** |  |
| There's a strength of team there. The relationship between school-based health practitioners and our school nurses, their ability to collaborate on immunizations, and all of those preventative practices leads to an overall healthier child. | School |
| Certainly an excellent relationship with the school nurses. They can drive or kill a program. | SBHC |
| **Coordinate with teachers, social workers and administration:** |  |
| They [SBHCs] have a table in front [of the opening day of school], and they're trying to get enrollment right out of the gate. | School |
| We have social workers in some of our schools... and we would communicate to help this one child. She's actually on a kidney transplant list now. And really, that particular social worker helped to make sure she got up to appointments. | Bassett |
| We can work with teachers, counselors. Really, teachers can make all kinds of observations that they're usually very willing to share with us. | SBHC |
| They're [school administration] great about putting up an occasional Facebook post on the district website that says “it's summertime, but we're still open or it's flu season, go get your flu shot with school-based health.” | SBHC |
| **Coordinate with parents and families** |  |
| Service that it [SBHC] provides our families keeps parents at work… They help with scheduling appointments outside of school. They help with making those connections for specialists. They help with insurance and walking families through that… they work with our families every step of the way. | School |
| [SBHCs and social workers] are working together to make sure dad was educated in her [SBHC patient] special renal diet. | Bassett |
| **Theme 1c: SBHCs Fit the PCMH Model: Continuous and Compassionate Care** | Source |
| **Children advocate for their own healthcare needs:** |  |
| We'll get high school students who can advocate for themselves… they can actually go to the clinic and say, ‘Can I see someone? I’m not feeling well’ versus having to utilize a parent. | School |
| With access in schools, kids have the ability to have conversations with a healthcare provider, one-on-one that they may be uncomfortable having with a parent present. | Bassett |
| It’s teaching good healthcare to kids like at a young age… presenting them with the ability to be good advocates for their own healthcare and seek out services when they need them. | SBHC |
| **Build a culture of health:** |  |
| The fact that these kids are getting care and setting the footprint for good preventative care is just going to make them healthier as adults. | Bassett |
| You know, [SBHC] patients who are hopefully at a greater likelihood to take care of their overall health as they get older and become adults. | SBHC |
| **True PCMH model:** |  |
| Honestly, I think that the school based health centers do it best...we call it the true PCMH model, meaning that you have behavioral health, dental, [and] primary care in one location. | Bassett |
| School-based health centers are sacred ground for kids...So kids tell us everything I mean, much more than their parents want to tell us sometimes. | Bassett |
| I asked every staff who interacts with kids, take on 2-3 kids who need extra attention and make them your special kids…Many of our kids do not have caring adults in their households. I called it “the power of one safe caring adult.” | SBHC |
| **Theme 2: Privacy and Confidentiality Ensure Child Empowerment** |  |
| **Information-sharing boundaries between SBHCs and schools:** |  |
| So there are laws where, medically, they [SBHCs] can't share things about patients, and there are laws for school districts that we can't overshare. So the only time that there would be sharing, I think [is] when the health and safety of a student is at risk and we're needing to support each other with the information. So yes, I would say, there are natural built-in privacy laws for both sides, and I don't think that that barrier gets crossed. | School |
| They [Schools] are giving us information and we'll just say, we'll look into it...They [schools] don't have access to our records by any means… So, we are tight on confidentiality… More information is coming to us than leaving us. | SBHC |
| **Confidentiality concerns from parents:** |  |
| …for parents, maybe, who are not running their household in the way that they should, there’s concern that schools are going to find out information through the clinic about them, and make judgments or treat them or their children differently. | School |
| **Privacy can deter some parents from enrolling their children in SBHCs:** |  |
| Contraceptives is a piece that's part of comprehensive health for teens and not all of our families agree with that. So I know that some families have not joined purely for that reason. | School |
| They [parents] don't want them [their kids] to receive those services without them [parents] knowing. | SBHC |
| **Theme 3: Challenges in SBHCs serving as PCMHs** | Source |
| **SBHCs Restricted from Serving Families:** |  |
| It'd be awesome if Bassett could see birth through adult. | School |
| More family-centered care… I would love to see evening hours for family therapy… that's what would serve families best. There's a lot of things that can't be solved by just talking with the child… The medical home model is family centered as well. So why not integrate the family into the care plan in general? | SBHC |
| **Loyalty to existing pediatricians is a primary barrier to SBHC enrollment** |  |
| Parents have a good relationship with a provider already, and they sometimes don't understand that it's okay to have more than one provider. | School |
| We have lower enrollment among younger children than we do older kids … Some of them feel loyal to their general practitioner…and only want to hear what that pediatrician has to say. | Bassett |
| I think people love their pediatrician…even though they never see him. Because the idea of doing that [switching to the SBHC] is sort of a betrayal. | SBHC |
| **Funding and resources:** |  |
| I would love for our State to provide incentives and funding for school based health across the board. | School |
| We need more help for our young people, especially coming out of COVID-19, so ideally a psychologist. | School |
| The space for them [doctors] to work and setup like a permanent space, instead of that portable equipment that they take everywhere for some schools. | Bassett |
| [SBHC] also goes a long way as far as retaining staff and recruiting staff… [who are] looking for a supportive place to work. | SBHC |
| **Innovative strategies:** |  |
| We send home a paper consent form… But most of the time they sit in the backpack… and never make their way back to us... It's time to send those electronically and allow the parents to sign them electronically. | SBHC |
| I would like to try and integrate more community services inside of our clinic. Bring the care to the patient.” | SBHC |

Source: Interviews with SBHCs in rural New York.

Note: School: school superintendent, Bassett: Bassett administrative employee, SBHC: SBHC medical provider
